# Genomic characteristics and profile of microsatellite primers for *Acanthogobius ommaturus* by genome survey sequencing

**DOI:** 10.1042/BSR20201295

**Published:** 2020-11-13

**Authors:** Bingjie Chen, Zhicheng Sun, Fangrui Lou, Tian-xiang Gao, Na Song

**Affiliations:** 1 Key Laboratory of Mariculture (Ocean University of China), Ministry of Education, Qingdao 266003, China; 2 Fishery College, Zhejiang Ocean University, Zhoushan 316022, China

**Keywords:** Acanthogobius ommaturus, GC content, Genome assembly, Genome size, SSR

## Abstract

*Acanthogobius ommaturus* is one of the suitable species to study the genetic mechanism of adaptive evolution, but there are few reports on its genetics. In the present study, the genomic survey sequencing method was used to analyze the genome characters of *A. ommaturus.* A total of 50.50 G high-quality sequence data were obtained in the present study. From the 19-mer distribution frequency, the estimated genome size was 928.01 Mb. The calculated sequence repeat rate was about 38.31%, the heterozygosity was approximately 0.17%, and the GC% content was approximately 40.88%. Moreover, 475,724 simple sequence repeats (SSRs) were identified. Among them, dinucleotide repeats were the most (53.70% of the total SSRs), followed by tri- (35.36%), hexa- (4.59%), tetra- (4.57%) and penta- (1.77%) nucleotide repeats type. This is the first genome-wide feature of this species to be reported.

## Introduction

*Acanthogobius ommaturus* belongs to the order Perciformes, Gobioidei, Gobiidae and *Acanthogobius*. It is a large-body goby fish distributed in the subsea or brackish waters of China, Korea, Japan and Indonesia [1]. The suborder Gobioidei is the largest group of fish species, with more than 2000 species reported [2]. Most goby fish have a wide distribution range, strong adaptability and high diversity, which are suitable for studying the genetic mechanism of adaptive evolution [3]. *Acanthogobius ommaturus* is an economically important species that has been reported to be the dominant species across its distribution area [4–6]. In recent years, many studies focusing on its ecological habits, morphological traits, biochemical determinations and feeding strategy in aquaculture industry have been conducted. For example, Feng et al. studied the morphological character, individual fecundity and ovary histology [1]. Huang et al. explored the effects of cryopreservation on several immune and antioxidative enzyme activities [7]. The characterization of Lactate Dehydrogenase in *A. ommaturus* was also analyzed [8]. Insoo and Hong investigated its feeding ecology in a tidal flat in Korea [9]. However, only few studies have involved its genetic diversity and phylogeographic patterns. Until now NCBI has only published the genome information of seven species of Gobioidei, and it cannot provide us valid information of *A. ommaturus* because of distinct genetic relationship between them [10]. In order to understand the genetic background of *A. ommaturus* and conduct further genetic studies, it is necessary to understand genome characteristics of *A. ommaturus*.

At present, high-throughput next-generation sequencing (NGS) is the main method of genome investigation, which is an important and effective strategy to generate genetic and genomic information [11]. Genome survey sequencing bases on the low-depth sequencing data of the small fragment library [12], and can effectively evaluate the genome size, GC content and heterozygosity through *K*-mer analysis [13,14]. Compared with traditional methods, it is also an effective method to characterize microsatellite markers of target species. As a codominant molecular marker, microsatellite marker has been widely used in studies related to genetic diversity and population structure [15,16]. Although there were some population genetic studies based on different molecular markers, such as mitochondrial DNA and AFLP [17,18], there were no studies on population genetics of *A. ommaturus* by using microsatellite markers. Therefore, in the present study, based on high-throughput sequencing, *K*-mer analysis was used to investigate the genomic characteristics of *A. ommaturus* and also give profile of its microsatellite primers. The results of the present study will provide theoretical basis for the subsequent development of the whole genome *de novo* sequencing assembly strategy [11,19], and can be used to describe the genetic background of many other marine animals [11,20–22]. In addition, the draft genome that is obtained by this sequencing is also conducive to the detecting of SNP loci.

## Materials and methods

We have read the policies relating to animal experiments and confirmed the present study complied (ARRIVE guidelines; EU Directive 2010/63/EU for animal experiments). All procedures performed in the present study were approved by the Institutional Animal Care and Use Committee of Ocean University of China.

### Sample preparation and DNA extraction

The samples were obtained by trawl net by fishermen from coastal water of Qingdao, China in October 2019, and we bought the lively and undamaged individuals in Qingdao wharf. Then, these fish were quickly transported to our laboratory for recovery. Approximately 7 days later, an active, healthy and undamaged *A. ommaturus* (overall length: 15.80 cm; body length: 12.9 cm; body weight: 30.50 g) was selected as the sample for sequencing. Selected *A. ommaturus* were killed to death with an overdose of anesthetic (tricaine methanesulfonate, MS-222, 100 mg/l). Subsequently, the fish were rapidly dissected, and part of the muscle tissues on the caudal peduncle were collected and preserved in 95% alcohol in ice box. The tissue samples were then quickly transformed to our lab where the samples were stored in −80°C until analysis. The traditional phenol–chloroform method was used to extract genomic DNA. The total DNA was treated with RNase to obtain DNA with high purity and without RNA pollution.

### Library construction and sequencing

After DNA extraction, library construction and sequencing were conducted. The qualified genomic DNA was broken to the target fragment (350 base pairs) by physical fragmentation (ultrasonic vibration), and then the small-fragment sequencing library was constructed through the steps of terminal repair, addition of A, addition of connector, selection of target fragment and PCR. Agilent 2100 and Q-PCR were used to detect the size of library fragments and library quantification to determine whether the library meets the sequencing standard. The library was fixed to the sequencing chip by bridge PCR. After library quality inspection and chip fixation, two paired-end DNA libraries were constructed with insert size of 350 base pairs, and then sequenced using the Illumina HiSeq X ten platform following the manufacturer’s protocol. The data generated by sequencing was used for the next step of bioinformation analysis after quality control. Entire read sets were deposited in the short read archive (SRA) databank (http://www.ncbi.nlm.nih.gov/sra/) and are available under accession number PRJNA658176.

### Analysis method

After GC distribution statistics and evaluation of quality values Q20 (ratio of bases with sequencing quality value above 20) and Q30 (proportion of bases with sequencing quality values above 30), clean reads were obtained after filtering the data of paired-end sequencing, which were used for a *K*-mer analysis. The *K*-mer analysis can be used to obtain the genome length, heterozygosity and repeat content.

The information of peak depth and number of predicted best *K*-mer could be obtained according to the results of the *K*-mer analysis, and subsequently this information was used to estimate the size of the genome. The formula was as follows in eqn (1):

(1) $$\text{Genome size }=\frac{\text{K-mer number}}{\text{K-mer peak depth}}$$

where *K*-mer number was the total number of predicted best *K*-mer, and peak depth was the expected value of the *K*-mer depth. The heterozygosity ratio and repeat sequence ratio were determined according to the methods described in Song et al. [23]. The software Jellyfish (v2.0, http://www.genome.umd.edu/jellyfish.html) and GenomeScope (v1.0, http://qb.cshl.edu/genomescope/analysis.php?code=example6) were used to conduct *K*-mer analyses [24,25]. The clean reads were assembled into contigs in software SOAPdenovo (v2.01, https://github.com/aquaskyline/SOAPdenovo2/) by applying the de Bruijn graph structure [26]. The paired-end information was then used to join the unique contigs into scaffolds [11]. To assess the completeness of the assembly, a Benchmarking Universal Single-Copy Orthologs (BUSCO, v.4.0, https://busco.ezlab.org/) evaluation was performed using the Eukaryota vertebrata_odb10 database [27]. The Perl script MISA was performed to identify microsatellite motifs in the *de novo* draft genome, and search parameters were set as minimum of 6, 5, 5, 5 and 5 repeats for detecting di-, tri-, tetra-, penta- and hexanucleotide microsatellite motifs, respectively. Primer3 (v2.3.7) software with standard parameters was used to identify the microsatellite loci [28,29].

## Results and discussion

### Genome sequencing and sequence quality estimation

A 350-bp insert library was constructed using the genomic DNA of the *A. ommaturus* sample, which was sequenced and filtered on the Illumina sequencing platform. A total of high-quality data of 50.50 Gb was obtained, with a total sequencing depth of approximate 54×. The Q20 and Q30 values were over 96% and 90%, respectively.

### Genome size and repeat ratio, and heterozygosity assessment

**Figure 1:**
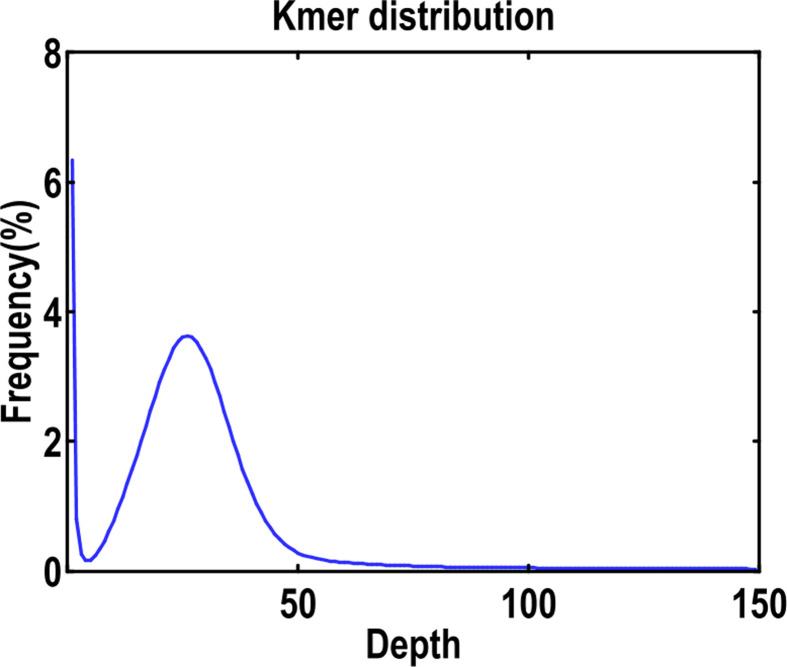
*K*-mer (*K* = 19) analysis for estimation of the genome size of *A. ommaturus* The *X*-axis was depth and the *Y*-axis was the proportion that represented the frequency at that depth.

Genome survey sequencing yielded a total of 50.50 Gb high-quality data, which were used for subsequent *K*-mer analysis. Using a 350-bp library data to construct a *K*-mer distribution map of *K* = 19 (Figure 1), and the genome size, repeat sequence ratio and heterozygosity were evaluated. The average *K*-mer depth that corresponded to the main peak, was 44, and the total number of *K*-mers obtained from the sequencing data was 44,434,973,013. After removing the anomalies of depth, a total of 41,080,854,391 *K*-mers were used for genome length estimation.

The calculated genome length was about 928.01 Mb. According to the reported genome data, the genome size of most fish is generally small and less than 1 Gb, except for a few fish species such as *Cyprinus carpio, Megalobrama amblycephala* and *Sinocyclocheilus graphami* [30]. Moreover, very few fish, like *Salmo salar* [31] and *Oncorhynchus mykiss* [32], are around 1.9-3.0 Gb because of a round of full gene duplication and the expansion of repeat sequence. The genome sizes of four Mudskippers (Gobioidei) were 0.983, 0.806, 0.780 and 0.739 Gb for *Periophthalmodon schlosseri, Periophthalmus magnuspinnatus, Boleophthalmus pectinirostris* and *Scartelaos histophorus*, respectively [10]. In the present study, genome size of *A. ommaturus* was estimated to be 928.01 Mb (0.928 Gb), which was in accordance with most fish.

According to the *K*-mer distribution, it was estimated that the repeat sequence content was about 38.31%. Most teleost fish exhibit a low repeat content, with an average of less than 30% [33], such as *Gadus morhua* (25.4%) [34], *Gasterosteus aculeatus santaeannae* (25.2%) [35] and *Larimichthys crocea* (16.38%) [36]. A few fish, such as *Danio rerio* and *Salmo salar*, have higher repeat sequences, reaching 52.2% [33] and 60% [31], respectively.

In the present study, there was no obvious heterozygous peak. Overall, the heterozygosity was low, and the estimated heterozygosity was about 0.17%. According to the characteristics of fish genome, draft genome can be obtained by next-generation sequencing technology.

### Genome assembly and GC content

With 45, 55, 65 and 75 bp *K*-mers, *de novo* assembly was performed using all of the clean reads (Table 1). When 55 bp *K*-mer value was used, the length of N50 could get the maximum value. The BUSCO evaluation results showed a low score caused by lacking of sufficient genomic data of related species The intrinsic defects of the second-generation sequencing technology may be also one of the reasons for the poor assembly effect (Table 2). Genomic GC content had a significant effect on the randomness of second-generation genome sequencing. Too high (>65%) or too low (<25%) GC content will lead to sequencing bias and seriously affect the results of genomic analysis. By analyzing the sequencing data of the survey library, the GC content of *A. ommaturus* genome was approximately 40.88%, which was suitable for further analysis and genome assembly.

**Table 1:** Statistics of *A. ommaturus* assembled genome sequences

*K*-mer depth (bp)	Contigs					Scaffolds				
	Total length (bp)	Total number	Max length (bp)	N50 (bp)	N90 (bp)	Total length (bp)	Total number	Max length (bp)	N50 (bp)	N90 (bp)
45	705,413,224	1,522,798	35,678	1120	203	720,996,115	598,674	51,912	2458	436
55	765,231,759	1,421,279	43,980	1237	216	776,582,763	717,328	60,036	2528	343
65	819,722,014	1,395,163	40,568	1267	227	827,753,551	848,081	66,118	2443	301
75	861,431,452	1,387,872	54260	1282	236	867,370,160	944,567	67,355	2343	283

**Table 2:** A assembly evaluation summary by BUSCO

Term	Results
Complete (C)	1300 (38.76%)
Complete (C) and single-copy (S)	1285 (38.31%)
Complete (C) and duplicated (D)	15 (0.45%)
Fragmented (F)	1150 (34.29%)
Missing (M)	904 (26.95%)
Total	3354

### Identification of SSR

Based on the genome survey sequence, the total number of identified microsatellite or simple sequence repeats (SSRs) was 349,138 (Table 3). Among them, 89,834 sequences contained more than one microsatellite site. The dinucleotide repeats were dominant (53.70%), followed by trinucleotide repeats (35.36%), hexanucleotide repeats (4.59%), tetranucleotide repeats (4.57%) and pentanucleotide repeats (1.77%). According to the distribution and frequency of microsatellite motifs, the dinucleotide repeats had the highest number and type of repeats, which was similar to the previous studies of other fish, such as *Pseudosciaena crocea* [37] and *Megalobrama amblycephala* [38]. The frequency of repetitions decreases exponentially with the length of the repetitions, because long mutations have high mutation rates [39]. This is consistent with the result that the number of repetitions is inversely proportional to the length of repetitions reported by Chen et al. (2010) [40].

**Table 3:** Simple sequence repeat (SSR) distribution statistics for *A. ommaturus*

Statistics	Di-	Tri-	Tetra-	Penta-	Hexa-
SSR number	187,501	123,452	15,969	6,182	16,034
Percentage	53.70%	35.36%	4.57%	1.77%	4.59%

**Figure 2:**
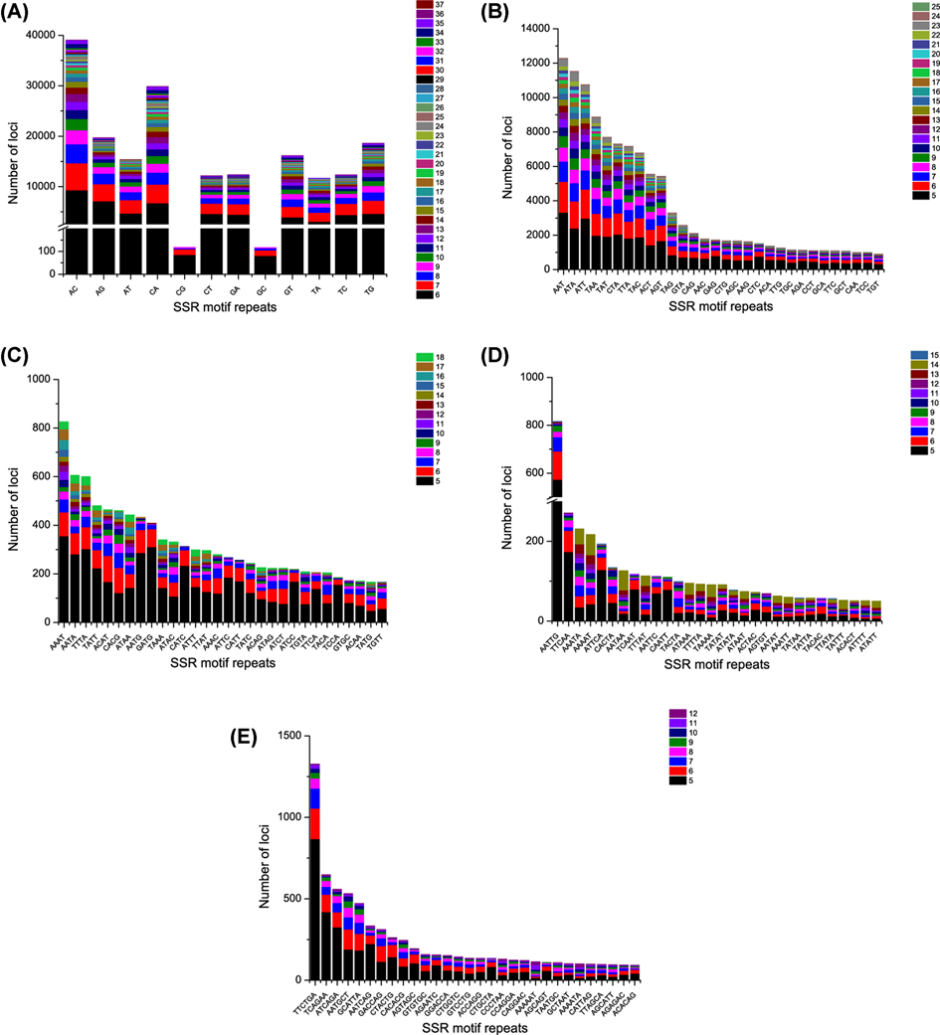
The distribution and frequency of microsatellite motifs (**A**) Frequency of different dinucleotide microsatellite motifs. (**B**) Frequency of different trinucleotide microsatellite motifs. (**C**) Frequency of different tetranucleotide microsatellite motifs. (**D**) Frequency of different pentabase microsatellite motifs. (**E**) Frequency of different hexanucleotide microsatellite motifs.

The AC repeat motif was the most frequent among all twelve types of dinucleotide repeat, whereas GC was the least frequent (Figure 2A). The AAT repeat motif was the most frequent among all sixty types of trinucleotide repeat (Figure 2B). The AAAT repeat motif was the most frequent among all types of tetranucleotide repeat (Figure 2C). The AATTG repeat motif was the most frequent pentanucleotide repeat (Figure 2D), and the TTCTGA was the most frequent hexanucleotide repeat (Figure 2E). There were only eight types of repeats detected in hexanucleotide repeats, and the number of repeats detected in dinucleotide repeats, trinucleotide repeats, tetranucleotide repeats and pentanucleotide repeats were higher than 10, even up to 35.

To provide more accurate information for SSR primer verification in future research, we selected 4996 pairs of sequences suitable for synthesizing microsatellite primers from the 89,834 sequences, with the following criterions: repetition times of SSR units are more than 6; length of SSR units is 3-10 bp; expected length of PCR products is 130–300 bp; primers that repeat the same four bases continuously are excluded. The selected SSR primers information were provided in the Supplementary Material.

## Conclusions

In summary, for *A. ommaturus*, the genome size was approximately 928.01 Mb, the proportion of repeats was approximately 38.31%, the heterozygosity rate was approximately 0.17%, and the GC content of the genome was approximately 40.88%. From the perspective of the basic structural characteristics of the genome, it was a simple genome. This paper could be conductive to the construction of subsequent fine map of the genome.

## Data Availability

All research data are included in the main text of the manuscript. Entire read sets were deposited in the short read archive (SRA) databank (http://www.ncbi.nlm.nih.gov/sra/) and are available under accession number PRJNA658176.

## Abbreviations

NGS: next-generation sequencing
SRA: short read archive
SSR: simple sequence repeat
